# Mouse H6 Homeobox 1 (*Hmx1*) mutations cause cranial abnormalities and reduced body mass

**DOI:** 10.1186/1471-213X-9-27

**Published:** 2009-04-20

**Authors:** Robert J Munroe, Vinay Prabhu, Greg M Acland, Kenneth R Johnson, Belinda S Harris, Tim P O'Brien, Ian C Welsh, Drew M Noden, John C Schimenti

**Affiliations:** 1 Department of Biomedical Sciences, College of Veterinary Medicine, Cornell University, Ithaca, New York 14853, USA; 2 The Jackson Laboratory, Bar Harbor, Maine 04609, USA

## Abstract

**Background:**

The H6 homeobox genes *Hmx1*, *Hmx2*, and *Hmx3 *(also known as *Nkx5-3*; *Nkx5-2 *and *Nkx5-1*, respectively), compose a family within the NKL subclass of the ANTP class of homeobox genes. Hmx gene family expression is mostly limited to sensory organs, branchial (pharyngeal) arches, and the rostral part of the central nervous system. Targeted mutation of either *Hmx2 *or *Hmx3 *in mice disrupts the vestibular system. These tandemly duplicated genes have functional overlap as indicated by the loss of the entire vestibular system in double mutants. Mutants have not been described for *Hmx1*, the most divergent of the family.

**Results:**

Dumbo (*dmbo*) is a semi-lethal mouse mutation that was recovered in a forward genetic mutagenesis screen. Mutants exhibit enlarged ear pinnae with a distinctive ventrolateral shift. Here, we report on the basis of this phenotype and other abnormalities in the mutant, and identify the causative mutation as being an allele of *Hmx1*. Examination of dumbo skulls revealed only subtle changes in cranial bone morphology, namely hyperplasia of the gonial bone and irregularities along the caudal border of the squamous temporal bone. Other nearby otic structures were unaffected. The semilethality of *dmbo/dmbo *mice was found to be ~40%, occured perinatally, and was associated with exencephaly. Surviving mutants of both sexes exhibited reduced body mass from ~3 days postpartum onwards. Most dumbo adults were microphthalmic. Recombinant animals and specific deletion-bearing mice were used to map the *dumbo *mutation to a 1.8 Mb region on Chromosome 5. DNA sequencing of genes in this region revealed a nonsense mutation in the first exon of H6 Homeobox 1 (*Hmx1*; also *Nkx5-3*). An independent spontaneous allele called misplaced ears (*mpe*) was also identified, confirming *Hmx1 *as the responsible mutant gene.

**Conclusion:**

The divergence of *Hmx1 *from its paralogs is reflected by different and diverse developmental roles exclusive of vestibular involvement. Additionally, these mutant *Hmx1 *alleles represent the first mouse models of a recently-discovered Oculo-Auricular syndrome caused by mutation of the orthologous human gene.

## Background

Homeobox proteins, characterized by the presence of a conserved 60 amino acid homeodomain, are encoded by a large group of genes that have diverse functions in development. Humans have about 235 functional homeobox genes that have been categorized into 11 major classes representing 102 families [1]. The ANTP (Antennapedia) class contains the highly studied HOX-like (HOXL) subclass of genes that are best known for their roles in specifying body plan organization from flies to mammals. Also well studied is the PAX gene family within the PRD (Paired) class, which generally functions to link spatial determination with lineage specification and differentiation. Mutations in genes from these and other classes have been linked to various human congenital disorders, including synpolydactyly, optic nerve colobomata, renal anomalies, mental retardation, Waardenburg's syndrome, neurological disorders, and cancer [2-4].

The H6 homeobox genes *Hmx1*, *Hmx2*, and *Hmx3 *(also known as *Nkx5-3*; *Nkx5-2 *and *Nkx5-1*, respectively), compose a family within the NKL subclass of the ANTP class of homeobox genes [1]. Hmx gene family expression is primarily limited to sensory organs, branchial (pharyngeal) arches, and the rostral part of the central nervous system [5]. Mouse *Hmx2 *and *Hmx3 *have identical expression patterns in the developing CNS and inner ear, except that in the latter, *Hmx3 *transcription in the otic epithelium initiates slightly earlier [6]. Targeted mutation of either gene in mice disrupts the vestibular system. Mutation of *Hmx3 *causes complete loss of the horizontal semicircular canal cristae and fusion of the utricle and saccule [7]. Ablation of *Hmx2 *is more severe, disrupting overall inner ear morphogenesis [6]. Absence of both *Hmx2 *and *Hmx3 *results in loss of the entire vestibular system [8]. Overall, the data suggest that these 2 genes have functional redundancy in those compartments of the inner ear in which expression overlaps [9]. Additionally, epistasis analysis indicates that these genes have interchangeable function in the hypothalamic/pituitary axis, demonstrating redundancy at the protein level. Their tandem organization on mouse Chr 7 and overlapping expression patterns raise the possibility that *Hmx1 *and *Hmx2 *share regulatory sequences [9]. The third member of the Hmx family, *Hmx1*, is located on Chr 5. It may have separated from the ancestral paralog of *Hmx2/3 *by a translocation event early in the vertebrate lineage.

Paralleling its sequence divergence and long-term physical separation from its paralogs, *Hmx1 *has a distinct expression pattern in neural crest derivatives. *In situ *hybridization studies found that at e10.5, *Hmx1 *is expressed in the eye, trigeminal cranial nerve, caudal-ventral region of the second branchial arch, sympathetic nerve ganglia, and dorsal root ganglia [5]. There is high expression in the neural retina and ear pinna. The expression patterns led Yoshiura et al to hypothesize the *Hmx1 *deficiency would lead to craniofacial, eye, and external ear defects [5]. Recently, a mutation in *HMX1 *(*NKX5-3*) was found to be responsible for Oculo-Auricular syndrome in three members of a consanguinous family [10].

In previous work, a semilethal mutation called *dumbo *was isolated in phenotype-driven screen for ENU-induced mutations on proximal mouse Chr 5 [11]. Affected animals exhibited laterally protruding ears that engendered the mutation's name. Here, we report that mutations in the homeobox-containing gene *Hmx1 *are responsible for the dumbo phenotype. Furthermore, we characterize effects of the mutation on postnatal growth, cranial development, the eye, and pre/perinatal lethality.

## Results

### Characterization of dumbo phenotype

**Figure 1:**
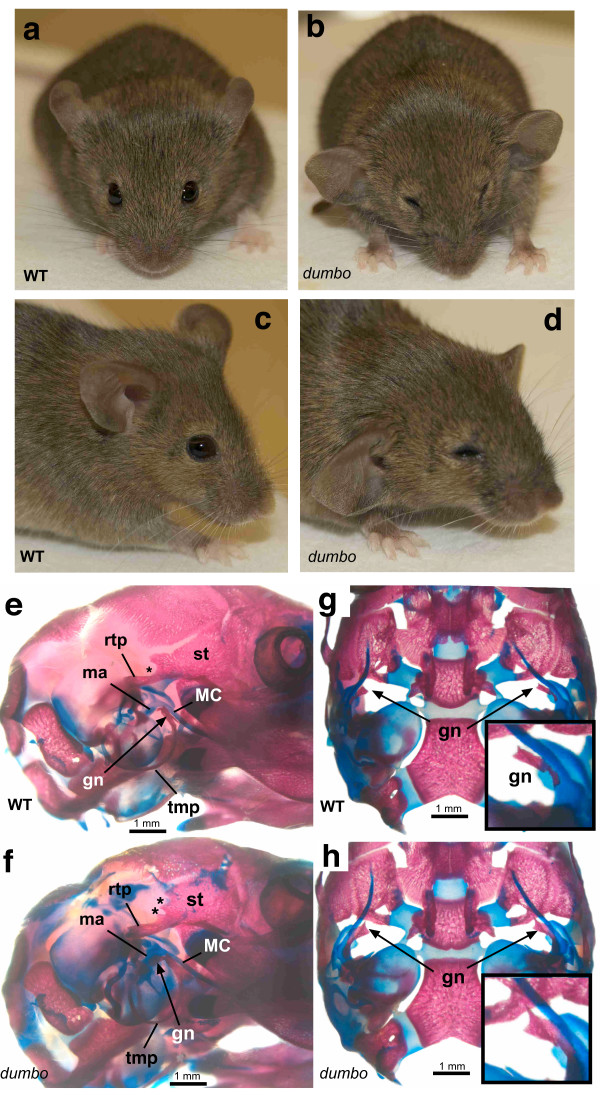
**Dumbo ear and cranial malformations**. Control (a, c) and dumbo (b, d) live mouse images. Note ventrolateral displacement of ear pinnae in the mutant. Image analysis of dissected control and dumbo pinnae revealed no difference in overall surface area, but the mutant ears were less concave, giving the impression of a larger area (data not shown). Most dumbo mice exhibit microphthalmia, as is evident in these images. Lateral (e, f) and dorsal (g, h) views of bone/cartilage skull preps. The calvaria was removed for the dorsal views. Compared to heterozygous controls (e), dumbo skulls (f) show malformations of the squamous temporal bone ("st"; the asterisk marks a region where dumbo mice exhibit an extra notch). Hyperplasia of the gonial bone ("gn") is evident in mutants (f, h) vs controls (e, g). rtp = retrotympanic process; MC = Meckel's cartilage; ma = malleus; tmp = ectotympanic.

As Wilson et al [11] previously reported within their supplemental data, dumbo (*dmbo*) homozygotes display laterally-protruding ears (Fig. 1a–d). To further assess skeletal anatomy, alcian blue/alizarin red staining of newborn skulls was performed. Comparison of mutants (Fig. 1f, h) to controls (Fig. 1e, g) revealed only minor differences. All dumbo mice (N = 9 vs. 6 controls) exhibited hyperplasia of the gonial bone (Fig. 1h), which in some cases extended rostrally to underlie the caudal border of the lamina obturans (middle portion of the future alisphenoid). The nearby proximal mandibular (Meckel's) cartilage, malleus, and tympanic ring (ectotympanum) were normal, as were the other middle ear ossicles. The size and position of the external acoustic meatus were normal in all animals examined.

The retrotympanic process of the squamous temporal bone was also enlarged. Immediately dorsal to this process in normal mice is a deep concave notch (asterisk, Fig. 1e). In 8/9 *dmbo *homozygotes, this caudal border of the squamous temporal bone had either two smaller notches (N = 3; asterisks, Fig. 1f), or other deformations of this region such an abnormally small notch (not shown). No defects in branchial arch's two skeletal structures, including the stapes, were detected.

**Figure 2:**
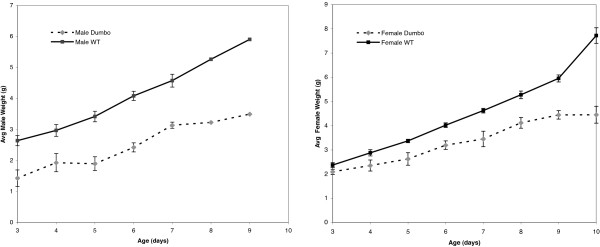
**Decreased body mass of dumbo mice after birth**. The error bars represent standard error of the mean.

In addition to the displaced ear pinnae, dumbo homozygotes appear smaller than their heterozygous littermates. To quantify this difference and determine its onset, *dmbo/Rw *heterozygotes were intercrossed, and the weights of progeny were recorded daily for 7–8 days beginning at postnatal day 3. *Rw *is a recessive lethal inversion with a dominant white spotting phenotype, allowing visual detection of genotypes from 3 days of age. It also serves as a balancer that prevents recombination between the *Dmbo*-containing chromosomes and the corresponding ~30 MB region spanned by *Rw*. As shown in Fig. 2, male homozygotes weighed about 50% less than heterozygotes at day 3, and continued to lag through day 9. Females were also smaller, but to a lesser degree (Fig. 2). To explore whether the weight differences were due to the mutants being outcompeted by their wild-type siblings for access to the mother, we also weighed animals produce by intercrosses of homozygotes (*dmbo/dmbo*). These animals had weights similar to the homozygotes produced from het × het intercrosses (data not shown), indicating that the reduced weight is an intrinsic characteristic. Whole animal necropsies of adults showed no recognizable differences between mutant and wild type mice, including the intestinal tract (not shown).

Breeding data of congenic animals confirmed the qualitative findings of Wilson et al [11] that *dmbo *is semilethal. Litters produced in *dmbo/Rw *× *dmbo/dmbo *crosses had 41% fewer *dmbo/dmbo *animals at three weeks of age (time of wean) than expected for Mendelian transmission (42 *dmbo/dmbo *of 142 total offspring from 33 litters; Chi^2 ^= 23.7, *P *< 0.0001). To determine if the loss of homozygotes occurred pre- or postnatally, we mated *dmbo/Rw *to *dmbo/dmbo *animals and monitored 11 litters during the first three days post-partum. Twelve of the 65 pups in these litters were found dead and 3 additional pups went missing. These 3 were eliminated from statistical analyses since their genotypes were unknown. Eleven of the 12 mice that died were genotyped as being *dmbo/dmbo *(Chi^2 ^= 8.33; P = 0.004). These data show that dumbo mice are much more likely to die prematurely than their unaffected littermates, and suggests that the shortfall of *dmbo/dmbo *at wean is probably due to peri- or postnatal lethality rather than embryonic lethality. To identify possible visible defects at birth, 3 litters produced from homozygous dumbo parents were observed either at the time of delivery or shortly thereafter. Seven of the 24 pups (29%) were born dead, of which 6 displayed exencephaly.

### Positional cloning reveals that dumbo is an allele of *Hmx1*

**Figure 3:**
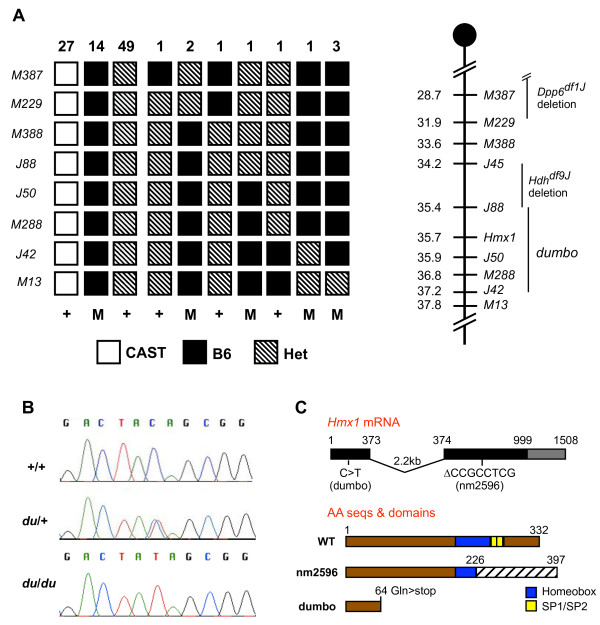
**Positional cloning of dumbo**. (A) Genetic mapping of *dmbo*. The left panel depicts the phenotypes ("+" = normal; "M" = mutant) and genotypes of 100 animals at the indicated loci (an "M" prefix = *D5Mit*; "J" = *D5Jcs*) produced from intercrosses of *dmbo*/+^*CAST *^mice. The right panel shows the locations of critical molecular markers on proximal Chr 5 (centromere on top), the locations of two deletions used for complementation analyses, and the location to which *dmbo *was mapped based on the combination of meiotic and deletion mapping. (B) Identification of a point mutation in the *dmbo *allele of *Hmx1*. Sequence traces are shown. (C) *Hmx1 *genomic structure and predicted protein products of wild type and mutant alleles. As indicated, the *dmbo *mutation causes premature termination of HMX1, whereas the *mpe *mutation potentially encodes 226 amino acids of the 332 amino acid HMX1 protein, but the frameshift appends 171 unrelated aa residues.

A combination of deletion and recombination mapping was used to map the dumbo mutation. Complementation crosses between dumbo homozygotes and the *Hdh*^*df*9*J *^or *Dpp6*^*df*1*J *^deletions [12] in *trans *to the *Rw *failed to yield a dumbo phenotype in the non-*Rw *progeny, thus demonstrating that these deletion regions (Fig. 3) do not harbor *dmbo*. Recombination mapping was then performed (see Methods), which localized *dmbo *to a 1.8 Mb region immediately distal to the distal *Hdh*^*df*9*J *^deletion breakpoint on Chr 5 (Fig. 2), defined proximally by *D5Jcs88 *at 35.47 Mb (July 2007 mouse genome assembly) and distally by *D5Jcs42 *at 37.39 Mb.

The *dmbo *critical region contains 20 annotated RefSeq genes. One of these, *Hmx1*, was deemed a potential candidate due to its narrow expression pattern that implied roles in craniofacial development and the ear pinna [5]. Sequence analysis (Fig. 3b) of multiple dumbo mutants and heterozygotes revealed a C→T mutation corresponding to amino acid 193, changing GLU65 to an amber stop codon (CAG→UAG). Accordingly to mouse nomenclature, we now refer to this mutant allele as *Hmx1*^*dmbo*^.

Confirmation that the *Hmx1 *mutation was causative for the dumbo phenotype came from complementation analyses with a spontaneous mutant called "misplaced ears" (*mpe)*, which arose in a C3H/HeJ colony of mice at The Jackson Laboratory. Homozygotes have an identical external ear phenotype, there is a shortfall of homozygotes from intercrosses (36/187; expected = 46.75; *P *= .0695), and the *mpe *mutation mapped to the same location on Chr 5 as *dmbo *(data not shown). Matings between mice carrying the two recessive mutations produced dumbo-like progeny. Sequencing of the *Hmx1*^*mpe *^allele revealed an 8 bp deletion in the region of exon 2 (nucleotides 674–681 of GenBank reference sequence NM_010445) encoding the homeodomain (not shown). This resulting frameshift beginning at amino acid 226 is predicted to replace the C-terminal 106 aa with an anomalous 171 aa (Fig. 3c).

### Transcription of *Hmx1 *in mutant embryos

**Figure 4:**
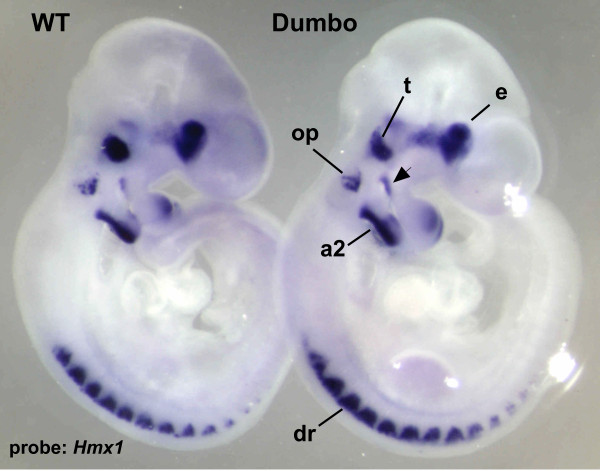
***Hmx1 *transcription in e10.5 day embryos**. Whole mount RNA *in situ *hybridization (at 10.5× magnification) of an *Hmx1 *antisense riboprobe (see Methods) to a heterozygous (WT) embryo and *dmbo/dmbo *embryo is shown. E = eye; t = trigeminal ganglion; op = otic placode; a2 = second branchial arch; dr = dorsal root ganglion. The arrowhead indicates mesenchymal expression in the proximal region of 1^st ^branchial arch caudal margin.

To confirm earlier studies on the expression pattern of *Hmx1 *during development [5], and to determine if the *Hmx1*^*dmbo *^mutation alters the pattern of *Hmx1 *expression, whole-mount RNA *in situ *hybridization was performed on wild-type and *Hmx1*^*dmbo*/*dmbo *^e10.5 embryos (Fig. 4). The staining patterns of both genotypes closely paralleled those obtained by Yoshiura *et al*, with prominent labeling of the developing eye, trigeminal ganglion, second branchial arch, and dorsal root ganglia. In addition, there was a band of labeled mesenchyme proximally (dorsally) along the caudal margin of the first branchial (pharyngeal) arch, which corresponds to the location at which progenitors of the gonial bone as well as the malleus are located. Finally, there was marked staining of the otic placode. The identical staining pattern between WT and homozygous mutants indicates that the nonsense mutation does not affect message stability. There were no discernable morphological defects related to the robust 2^nd ^branchial arch expression site.

### Hearing and eye morphology in Dumbo mice

Because *in-situ *hybridization data showed *Hmx1 *expression in the otic placode, and disruption of its paralogs *Hmx2 *and *Hmx3 *resulted in hearing loss, we tested hearing acuity in control and mutant animals. Both *Hmx1*^*dmbo*^/*Hmx1*^*dmbo *^and heterozygous siblings displayed a normal Preyer response in response to high frequency sound bursts in a click box test. Additionally, auditory brainstem response (ABR) tests (Table 1) found no threshold increases in mutants compared to controls, demonstrating that hearing in dumbo mice is normal despite ear pinna dysmorphology. Furthermore, dumbo mice can swim, indicating an absence of vestibular defects.

**Table 1:** Auditory Brainstem Response (ABR) data

		ABR thresholds			
		
Genotype*	Sex	Click	8 kHz	16 kHz	32 kHz
*dmbo/dmbo*	F	50	45	25	55
*dmbo/dmbo*	M	55	45	25	45
*dmbo/dmbo*	M	45	55	25	70
*Rw/dmbo*	M	45	30	20	70
*Rw/dmbo*	M	45	35	30	55
*Rw/dmbo*	M	50	50	45	95

* All mice were littermates and 37 days old when tested

*Hmx1 *is highly expressed in the developing eye, and humans with mutations in the orthologous *HMX1 *(*NKX5-3*) gene exhibit a variety of ophthalmic anomalies including microphthalmia, anterior segment dysgenesis, cataracts and colobomata [10]. As shown in Fig. 1, gross inspection suggested that most dumbo mice were microphthalmic. Clinical ophthalmological examination (indirect ophthalmoscopy, slit lamp biomicroscopy) of 2 affected mice confirmed that both were bilaterally microphthalmic, with low grade keratoconjunctivitis sicca ("dry eyes") and entropion. The latter two defects were presumed to be secondary to the microphthalmia. Otherwise the eyes were clinically normal; specifically, no evidence of microcornea, anterior segment dysgenesis, cataract, colobomata, retinal dysplasia or retinal detachment was detected.

## Discussion

The skeletal analyses we performed suggest a possible basis for the external ear phenotype of dumbo mice. The major defects we noted were enlargement of the gonium (gonial bone) and malformations of the caudal border of the squamous temporal bone. Both are in proximity to the external ear, and thus may influence the situation of the pinna, although the position of the external acoustic meatus was unchanged. Alternatively, as predicted by Yoshiura et al [5], the expression of *Hmx1 *in the pinna itself might be entirely responsible for the dysmorphology. If this is the case, then the consequences of the bone malformations remain uncertain. It is possible that the phenotype is a consequence of these combined factors.

The gonium forms by intramembranous ossification and helps anchor the malleus to tympanic ring, which later forms the outer wall of the middle ear. While many mutations cause dysmorphologies of the middle ear ossicles [13], we are unaware of mouse mutations that specifically cause an absence of the gonium. A *Bapx1 *knockout causes loss of the gonium along with hypoplasia of the tympanic ring. It also causes perinatal death [14]. Inactivation of only one copy of *Bapx1 *produces hypoplasia of the gonium but no detectable middle-ear phenotype [14]. Null mutations of *Dlx5 *and *Dlx6 *both cause the formation of an ectopic cartilaginous and intramembranous skeletal bridge that originates from the proximal mandibular (Meckel's) cartilage, at its junction with the malleus and extends medially to the pterygoid bone [15]. This bridge may partially or fully incorporate the gonial bone, which is often hypertrophic [16]. Reflecting their expression in all branchial arches, these mutations also cause growth reductions and dysmorphologies in the upper and lower jaws.

There is at present no obvious connection between the two morphological lesions that excludes effects on other nearby skeletal structures. Both the gonial and squamosal temporal bones arise within the proximal (dorsal) neural crest-derived mesenchyme of the first branchial arch. The precise location of these primordia relative to other first arch-derived otic structures has not been mapped in mammalian embryos. However, based on their homologies with structures in avian/reptilian species, one would surmise that the progenitors of the malleus and incus are interposed between those of the squamous temporal and gonial bones [17].

Previous studies suggested that the range of sound frequencies detectible by various species is related to the gonium-mediated degree of fusion between the malleus and the tympanic bone [18,19]. Since *Hmx1*^*dmbo *^causes hyperplasia of the gonium, we considered it possible that the range of detectable frequencies may be altered in these animals. However, the ABR data revealed no anomalies in hearing acuity across the frequency spectrum tested (Table 1). Like dumbo mice, people with an *Hmx1 *mutation were found to have normal audiograms and vestibular function [10].

At present, we do not understand the basis for the substantial perinatal lethality and reduced body mass of *Hmx1 *mutant mice, or if these two phenotypes are related. The RNA *in situ *hybridization studies revealed that the absence of functional HMX1 in dumbo embryos did not noticeably alter the level of *Hmx1 *transcription or perturb the developing structures in which *Hmx1 *is expressed during embryogenesis up to e10.5, suggesting that developmental homeostasis of these structures was not markedly disrupted. The smaller size of mutant mice and the observation (in one experiment) that 6/7 dumbo animals found dead at birth were exencephalic, are suggestive of a possible defect in cell proliferation at particular developmental stages or compartments. Interesting, mutation of the *C. elegans *Hmx ortholog, Mls-2, causes decreased proliferation and aberrant cell fate specification in the postembryonic mesodermal ("M") lineage [20]. As a homeodomain-containing protein that likely acts as a transcription factor, a key step in understanding the function of HMX1 will be to identify its gene targets in affected cell types.

Mutation of *HMX1 *(*NKX-5*) in humans causes an Oculo-Auricular syndrome. Dumbo mice substantially recapitulate the phenotypes of the three known human patients. As in mice, the human patients have external ear phenotype lobule deformations and aplasia. It is interesting to speculate whether polymorphisms in *HMX1*/*NKX-5 *might be related to variations in external ear phenotypes. Additionally, while none of the three human patients had other overt dysmorphologies, one had spina bifida occulta, which might be construed as a mild manifestation of the exencephalic dumbo pups we observed at birth.

Although *Hmx1 *is expressed in the eye lens and retina during development, and the HMX1-deficient humans have a variety of ocular defects (microcornea, microphthalmia, anterior-segment dysgenesis, cataract, colobomata, abnormalities of the retinal pigment epithelium, and rod-cone dystrophy) the defects in mice appear restricted to microphthalmia. Interestingly, morpholino knockdown of the zebrafish ortholog was found to cause microphthalmia [10]. The basis for the species differences might yield insight into the etiology of the human disorder. It is also important to note that strain background may influence the microphthalmia, since this phenotype was not as prevalent during early maintenance of dumbo mice [11]. It is possible that genetic heterogeneity also affects the severity of endophenotypes in HMX1-deficient humans.

## Conclusion

In mice, mutation of *Hmx1 *yields the distinguishing feature of ventrolateral displacement of ears, giving the appearance of its Disney character namesake. While the expression pattern of *Hmx1 *correlates with the ontology of defects in the ear pinnae, gonium, and squamous temporal bones, the relationship to semilethality and reduced body mass warrants further investigation. Finally, these mice provide the first model for a particular human Oculo-Auricular syndrome.

## Methods

### Mapping of dumbo to the *Hmx1 *locus

The *Hmx1*^*dmbo *^allele was induced by ENU mutagenesis of C57BL/6 (B6) inbred mice. It is maintained in a mixed B6 × C3HeB/FeJ (C3H) background, typically by intercrossing of *dmbo*/*Rw *animals. The visibly-marked, recessive lethal *Rw *inversion prevents the recovery recombinants across the ~50 Mb it spans on proximal Chr 5 [21], and these properties were essential in the isolation of ENU-induced mutants mapping to this region [11]. For recombination mapping of the mutation, *dmbo *was placed in *trans *to a *Mus castaneus *(strain CAST/EiJ) version of proximal chromosome 5, and heterozygotes (*dmbo*/+^CAST^) were intercrossed. The offspring (representing a total of 236 meioses) were scored for the dumbo phenotype, and PCR-genotyped with polymorphic microsatellite markers at proximal and distal ends of the *Rw *region. Recombinants were re-typed with additional markers to refine the locations of the crossovers (see Fig. 3). The primer sequences of all marker loci can be found in the Mouse Genome Database: .

Mice bearing the *dmbo *and *mpe *alleles are available from The Jackson Laboratory.

### Whole-mount RNA in situ hybridization to mouse embryos

A 535 bp fragment corresponding mostly to the 3' UTR of *Hmx1 *was amplified with the following primers: 5'-GCTTGCTTACCCGCTTGC-3' and 5'-GTCAAGCCAAGAGAACCAAGG-3'. The product was cloned into the plasmid vector pCRII-TOPO (Invitrogen), and used to generate digoxigenin-labeled antisense riboprobes. Embryos were dissected in cold PBS and immediately fixed in 4% paraformaldehyde overnight at 4°C followed by several rinses in PBST (PBS with 0.1% Tween-20), dehydration to 100% methanol and stored at -20°C. Whole mount *in situ *hybridization was performed as described [22]. In order to gain a qualitative assessment of relative expression levels, all WT and mutant tissues were processed in a single vial and developed for an identical length of time.

### Skeletal analyses

Newborn pups were sacrificed by decapitation, and the skulls were stained with alcian blue (for cartilage) and alizarin red (for bone) as we previously described [23]. Since dumbo pups are visually indistinguishable from heterozygotes at birth, a piece of tissue was used for genotyping. The DNA was PCR amplified with primers for *D5Mit13*, which is polymorphic between B6 (dumbo allele) and *Rw*.

### Hearing Tests

The click box and ABR tests were performed as previously described [11,24].

### Strain Availability

The strains B6;C3Fe-*Hmx1*^*dmbo*^/Rw/JcsKjn (Stock Number 8677) and C3H/HeJ-*Hmx1*^*mpe*^/J (Stock Number 8676) are available from The Jackson Laboratory.

## Authors' contributions

RJM genetically mapped and positionally cloned *Dmbo*, did the weight analyses, craniofacial preps, and participated in writing the manuscript. VP did sequencing to identify the *Dmbo *mutation, performed background research and writing on Hmx genes, and prepared the weight data for publication. GMA performed the eye analyses and interpretations and corresponding write-up. KRJ performed the hearing tests and analyses, and prepared the data and write-up for that part; BSH mapped *mpe*; TO mentored ICW and participated in manuscript preparation; ICW participated in the *in situ *hybridization experiments and interpretation thereof; DMN performed the anatomical analyses of the skulls; interpretation of the mutant phenotype, and co-writing of the manuscript; JCS supervised the overall study and wrote the majority of the paper. All authors read and approved the final manuscript.
